# Pyrolysis converts urban pruning waste into biochar with soil and climate benefits

**DOI:** 10.1038/s41598-025-32360-9

**Published:** 2025-12-22

**Authors:** Rafaela de Jesus Paula, Virgílio de Almeida Pereira, Fernando Lopes Latorre, Cinthia Oliveira de Souza Nogueira, Isabela Aroeira de Almeida, Pablo Aislan Freitas e Silva, Osania Emerenciano Ferreira, Robson Pereira de Lima, Augusto César da Silva Bezerra, Alan Rodrigues Teixeira Machado

**Affiliations:** 1 Departamento de Ciências Exatas, Universidade do Estado de Minas Gerais, João Monlevade, MG 35930-314 Brazil; 2 Waycarbon Soluções Ambientais e Projetos de Carbono S.A, Belo Horizonte, MG 30140-080 Brazil; 3 Latorres Consultoria Ltda, Sete Lagoas, MG 35702-882 Brazil; 4 Programa de Pós-graduação em Ciências Ambientais, Universidade do Estado de Minas Gerais, Frutal, MG 38202-436 Brazil; 5 Departamento de Ciências Agrarias e Biológicas, Universidade do Estado de Minas Gerais, Frutal, MG 38202-436 Brazil; 6 Departamento de Geociências, Ciências Humanas e Linguagens, Universidade do Estado de Minas Gerais, João Monlevade, MG 35930-314 Brazil; 7 Departamento de Engenharia de Transportes, Centro Federal de Educação Tecnológica de Minas Gerais, Belo Horizonte, MG 30421-169 Brazil

**Keywords:** Waste, Carbon, Soil, Conditioner, Carbon sequestration, Agriculture, Engineering, Environmental sciences

## Abstract

Urban pruning waste represents an environmental challenge for cities, and its valorization through biochar production in continuous reactors at the industrial scale has scarcely been explored. This study evaluated the conversion of residues in a horizontal pilot-scale continuous reactor. Four biochars (BC1–BC4) were produced under different operational conditions, including various particle sizes (< 10 to < 90 mm), inlet temperatures (140–300 °C), average operating temperatures (290–350 °C), and residence times (60–70 min). All the samples were characterized by their elemental composition (CHN/O), whereas BC4 was selected for more comprehensive analyses, including potentially toxic metals and organic contaminants, scanning electron microscopy with energy dispersive X-ray spectroscopy (SEM–EDS), surface area determination (BET), and thermogravimetric analysis (TGA) under air, and recalcitrance index (R_50_). The carbon sequestration potential was estimated from the C_org_ and H/C_org_ ratio. The biochars displayed distinct characteristics. BC1 and BC4 presented the highest carbon contents (71.63% and 70.80%, respectively) and the lowest H/C and O/C ratios. BC3 had the lowest carbon content (47.38%) and the highest ash fraction (38.61%). BC4 had a high degree of carbonization, low ash content (15.40%), favorable atomic ratio (H/C_org_ = 0.45; O/C_org_ = 0.13), alkaline pH (8.77), and low specific surface area (4.58 m² g^−1^). It also complied with European Biochar Certificate thresholds for metals and organic contaminants. Thermal analysis confirmed moderate recalcitrance (R_50_ = 0.5), and its estimated carbon sequestration potential was 1.62 t CO₂e t^−1^ biochar. Overall, urban pruning waste biochar has proven safe, stable, and potentially useful for soil improvement and climate change mitigation, although feedstock heterogeneity and logistics remain challenges for large-scale implementation.

## Introduction

The large-scale generation of waste from urban pruning, estimated at 47 kg per capita per year globally, represents a significant challenge for urban solid waste management^1^. Despite its volume and valorization potential, this biomass is often neglected and commonly landfilled, particularly in developing countries^2,3^. Its collection and use face logistical barriers such as geographic dispersion, seasonality, and the costs of shredding, drying, and transportation^4^. In Brazil, the National Solid Waste Policy (Law 12,305/2010) establishes guidelines for integrated waste management, emphasizing recycling and recommending landfills only as a last resort; however, more than a decade later, many municipalities still fail to comply^5^, and economic incentives for the energy recovery of urban pruning waste are scarce. As a result, resources with high potential for sustainable applications are underutilized. Given its composition, which is rich in cellulose, hemicellulose, and lignin, urban pruning biomass represents a promising feedstock for thermochemical conversion routes such as pyrolysis^6^.

In particular, pyrolysis decomposes biomass under nonoxidizing conditions and produces bio-oil, biochar, and noncondensable gases^7^. It can be classified into slow (or conventional), fast, and flash types, depending on the heating rate, residence time, and temperature^8^. Slow pyrolysis is typically conducted at 300–400 °C, with heating rates of 0.1–1.0 °C min^−1^ and residence times ranging from minutes to hours, resulting in yields of 30–40% biochar^9,10^. Fast pyrolysis takes place at 500–700 °C, with heating rates of 10–200 °C min^−1^ and short residence times of ~ 2 s, favoring bio-oil yields of 50–75%, alongside 12–25% biochar and 13–25% syngas^9,10^. In contrast, flash pyrolysis operates at 900–1200 °C, with heating rates above 1000 °C min^−1^ and residence times shorter than 1 s, generating 60–75% bio-oil, 12–20% biochar, and 13–20% syngas^9,10^.

Biochar produced from urban pruning waste has physicochemical properties such as increased pH, cation exchange capacity, electrical conductivity, porosity, and water retention in soil, as well as slow nutrient release^11,12^. These characteristics support its use in environmental applications, including the remediation of degraded areas, the improvement of soil quality in urban environments, and the control of compaction^13,14^. In addition to being used for soil management, biochar derived from this biomass has also demonstrated potential as an adsorbent for organic and inorganic contaminants, including potentially toxic metals and herbicides^11,15^. Its adsorptive capacity is strongly influenced by pyrolysis temperature, carbon content, and the presence of oxygenated functional groups, with studies reporting complete removal of Pb and Cd and up to 86.2% Mn at higher pyrolysis temperatures^11^.

Although biochar has promising applications, potential risks must be carefully considered. Ecotoxicological tests with isopods (*Porcellio scaber*) revealed behavioral and physiological changes after exposure to carbonaceous materials from urban pruning waste, underscoring the need for prior ecological safety assessments^16^. In addition, incorporating biochar into Oxisols has been shown to increase electrical conductivity and permeability, supporting geoenvironmental uses but also raising concerns about potential leaching^12^. These highly weathered tropical soils are characterized by high porosity, low bulk density, and a microgranular structure influenced by Fe and Al oxides^17^, conditions that may interact with biochar properties and affect its behavior in the soil environment. Apart from soil-related effects, practical challenges also emerge: the logistics of collecting and transporting pruning residues to centralized pyrolysis units can limit large-scale implementation, whereas the intrinsic variability of feedstocks leads to considerable heterogeneity in biochar quality. Addressing these challenges requires careful planning of biomass collection and transport. Moreover, detailed characterization of the material is essential to ensure safe and effective applications despite feedstock variability.

Despite progress in understanding the applications of biochar derived from urban pruning waste, most studies remain limited to laboratory settings and controlled conditions^18,19^. This limitation hinders the extrapolation of results to large-scale applications and restricts insights into pyrolysis performance under practical scenarios. Nevertheless, several pilot- and commercial-scale investigations have been conducted with different feedstocks, such as sewage sludge, food waste, tomato crop residues, digestates, and mixtures of biosolids with green waste, often integrating hydrothermal pretreatment, anaerobic digestion, and pyrolysis for combined energy recovery and biochar production^20–26^. Other studies have demonstrated the technical feasibility of scaling up biochar production from agricultural pruning residues to pilot reactors and even industrial reactors, highlighting the importance of controlling temperature and residence time to ensure biochar quality^27^.

Although previous studies have advanced the scaling up of biochar production from diverse organic residues, the specific case of urban pruning remains poorly explored under continuous, industrial-scale conditions. This knowledge gap limits the understanding of how particle size, reactor operation, and feedstock heterogeneity influence biochar yield, composition, and environmental safety. To address this issue, the present study evaluated the production of biochar from urban pruning waste in a horizontal continuous reactor. Specifically, this study aimed (1) to evaluate the influence of particle size on biochar yield and composition and (2) to estimate its carbon sequestration potential on the basis of elemental characteristics and stability indicators.

## Materials and methods

### Collection and preparation of urban pruning waste

Urban pruning waste was collected and supplied by ESAL Empreendimentos e Soluções Ambientais, located in Ribeirão Preto, São Paulo, Brazil. The waste included a heterogeneous mixture of branches, leaves, and tree trunks, which were arranged in piles on compacted soil in an unpaved yard. The company processes approximately 2,000 t of such residues per month. Shredding was carried out via a diesel-powered shredder (Tana Shark 400DT, Tana Oy, Jyväskylä, Finland), which was supported by a hydraulic excavator (Caterpillar 320, Caterpillar, Piracicaba, Brazil) for feeding and a wheel loader (Caterpillar 924k, Caterpillar, Piracicaba, Brazil) for material handling. Two target particle sizes were produced, corresponding to materials passing through 90 mm and 50 mm sieves, with average diesel consumption values of 4.5 and 5.5 L per processed ton, respectively.

After processing, contamination by soil and rock was observed, leading to the use of a rotary screen equipped with a 10 mm mesh sieve for separation. Two fractions were obtained: the oversize fraction (> 10 mm, retained on the sieve) and the undersize fraction (< 10 mm, passed through the sieve). Rocks were manually removed from the oversize fraction. Bulk density was determined as the ratio of the sample mass to the total volume it occupied by weighing the material in a container of known volume (200 L). The screened samples presented bulk densities ranging from 170 to 278 kg m^− 3^, reflecting the heterogeneity of the biomass and the presence of mineral matter. For the 90 mm material, only the oversize fraction (particle size, PS, in the range of 10–90 mm) was considered suitable for pyrolysis due to contamination by soil and rocks, whereas for the 50 mm material, both the oversize (10–50 mm) and undersize (PS < 10 mm) fractions were fed separately into the reactor. The oversize fraction had a bulk density of 174.1 kg m^− 3^, whereas the undersize fraction reached 262.9 kg m^− 3^, indicating greater contamination by soil in the finer fraction.

To reduce contamination by soil and rock, a second collection and processing step was performed. This time, the material was sent directly to the shredder, deposited on a paved surface, and then stored in bags. There was no contact between the loader and the ground or rocks, minimizing contamination. In this second collection, only the material passing through the 90 mm (PS < 90 mm) sieve was used.

### Pyrolysis of urban pruning in a horizontal continuous reactor

**Fig. 1:**
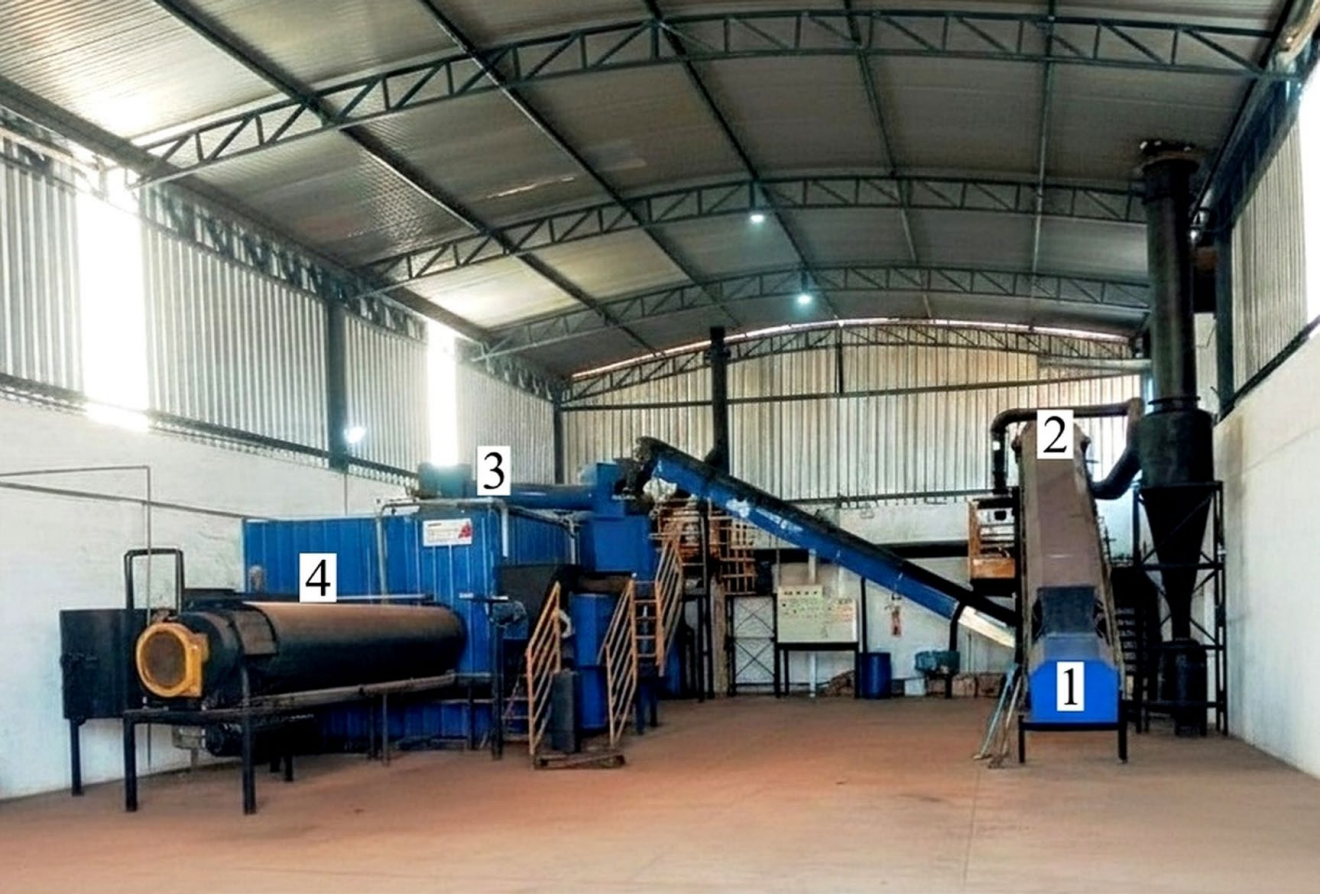
The system operates with continuous feed via a conveyor belt, integrating (1) a manual biomass feeding unit, (2) a drying section, (3) a pyrolysis reactor, and (4) cooling of the solid product.

The production of biochar samples was carried out in a horizontal continuous reactor, model LZ™ (MDL Ambiental/Latorres Consultoria, Dores do Indaiá, Brazil), with an estimated operational capacity of 20 to 25 tons of biochar per month. As a pilot-scale unit (Fig. 1), the reactor does not operate with automated temperature control, and the heat balance depends on both initial wood chip combustion and exothermic reactions from biomass decomposition, which may result in variations in operating parameters. The system integrates four main stages: drying of the biomass, pyrolysis, combustion of pyrolysis gases, and cooling of the biochar^28^. Biomass feeding into the system was performed continuously via a conveyor belt. Biomass drying was conducted in a rotary dryer coupled to the system, using the reactor’s exhaust gases as the heat source. The residence time of the biomass in the dryer was approximately 5 min, which allowed for a reduction in moisture content to approximately 8.5% ww^−1^.

The reactor was heated by burning eucalyptus wood chips, which provided the initial energy input and were occasionally supplemented during operation to sustain the required temperature. The initial heating of the system took 140 min until the operating temperature stabilized, while the cooling time of the biochar in the heat exchanger was approximately 100 min. From the first collection, three biochar samples were produced and coded BC1, BC2, and BC3. The BC1 sample was produced from 98 kg of urban pruned material (10–90 mm) inserted into the reactor at 140 °C. The average operating temperature was 300 °C, with a residence time of 70 min and a feed rate of approximately 1.0 kg min^−1^. The inlet and outlet gas temperatures were approximately 800 °C and 230 °C, respectively.

The BC2 sample was obtained from 71 kg of urban pruning (10–50 mm) and was processed at an inlet temperature of 300 °C and an average operating temperature of 290 °C. The residence time was approximately 60 min, with a feed rate of approximately 1.0 kg min^−1^. The combustion gases entered 700 °C and exited 180 °C. The BC3 sample was obtained from urban pruning residues with PS < 10 mm, totaling 118 kg of processed residues. The operating temperature was maintained at 300 °C for 60 min, with a feed rate of approximately 1.0 kg min^−1^. The inlet and outlet gas temperatures were 685 °C and 335 °C, respectively.

From the second collection, the BC4 sample was obtained from 567 kg of oversized urban pruning residues (PS < 90 mm) processed in the same continuous system. The average operating temperature was approximately 350 °C, with a residence time of 60 min and a feed rate of approximately 1.6 kg min^−1^. The inlet and outlet gas temperatures were approximately 820 °C and 425 °C, respectively. The main operational parameters for each sample are presented in Table 1.

**Table 1:** Operating parameters of the pyrolysis experiments for the biochar samples.

Sample	Collection	Particle size (PS) (mm)	Urban pruning waste (kg)	Inlet temperature (°C)	Average operating temperature (°C)	Residence time (min)
BC1	1st	10–90	98	140	300	70
BC2	1st	10–50	71	300	290	60
BC3	1st	< 10	118	300	300	60
BC4	2nd	< 90	567	190	350	60

1st and 2nd refer to the first and second urban pruning waste collections, respectively.

### Biochar sample characterization

The elemental composition was assessed via a PerkinElmer CHN/O 2400 analyzer (PerkinElmer, Inc., Waltham, MA, USA) to quantify the carbon (C), hydrogen (H), and nitrogen (N) contents, with analyses performed in duplicate. The ash content was determined using 1.0 g of dried and sieved sample (< 0.210 mm), calcined in a muffle furnace of around 9 L volume [35 cm (length) × 16 cm (width) × 16 cm (height)] at 700 ± 10 °C until complete combustion, with results expressed as the mean of duplicates. Additionally, BC4 was analyzed for further parameters in accordance with the European Biochar Certificate (EBC) guidelines^29^. Morphological and elemental composition analyses were performed by scanning electron microscopy (SEM) coupled with energy dispersive X-ray spectroscopy (EDS). A Vega 3 LMU microscope (Tescan, Brno-Kohoutovice, Czech Republic) with an X-MaxN detector (Oxford Instruments, Oxford, UK) was used. The biochar was mounted on stubs with carbon tape and coated with gold-palladium via an SC7620 sputter coater (Quorum Technologies, Ashford, UK). Images were obtained at an acceleration voltage of 15 kV.

To complement these analyses, the specific surface area (*S*_*BET*_) was determined via the Brunauer–Emmett–Teller (BET) multipoint method^30^. Nitrogen (N_2_) adsorption measurements were carried out with a surface area and porosity analyzer (Nova 600, Anton Paar GmbH, Graz, Austria). The pore size distribution was determined via nonlocal density theory (NLDFT) models. Thermal characterization was conducted via thermogravimetric analysis (TGA) via an STA 7300 analyzer (Hitachi, Tokyo, Japan). Approximately 10 mg of sample was heated from 25 °C to 1000 °C at 10 °C min^−1^ under a synthetic air atmosphere. The thermal recalcitrance index (R_50_) was determined from the air thermogram. After the moisture and ash contents were corrected, the temperature at which 50% mass loss occurred (T_50% biochar_) was identified. R_50_ was calculated via Eqs. (1) and (2) as described by Ganesan et al.^31^.

1 $$\:{R}_{50}=\frac{{T}_{50\%\:\:biochar}}{{T}_{50\%\:\:graphite\:}}$$

where T_50% biochar_ and T_50% graphite_ correspond, respectively, to the temperatures at which 50% mass loss occurs for biochar and graphite (886 °C).

2 $$\:{W}_{i,corrected}=100+\left[100\:\times\:\left(\frac{{W}_{i,\:\:uncorrected}-\:{W}_{200,\:\:uncorrected}}{{W}_{200,\:\:uncorrected}\:-{W}_{final,\:\:uncorrected}}\right)\right]$$

where W_i_, _corrected_ is the final corrected mass of the biochar; W_i, uncorrected_ is the initial mass at the beginning of the assay; W_200_,_uncorrected_ is the mass at 200 °C, the temperature at which free and nonstructural water is assumed to have been eliminated; and W_final, uncorrected_ is the mass at the end of the assay, when no further oxidation or mass loss occurs.

### Carbon sequestration potential (CSP)

The CSP of the urban pruning waste biochar (BC4) was estimated on the basis of a 100-year time horizon. For this purpose, Eqs. (3) and (4), adapted from Woolf et al.^32^, were used.

3 $$\:{CSP}_{{t}^{-1}}={F}_{C}\times\:{F}_{perm}\times\:\:\frac{44}{12}$$

where *CSPt*^−1^ represents the carbon sequestration potential, expressed in tons of CO_2_ equivalent per ton of biochar (t CO₂e t⁻¹ of biochar); FC corresponds to the mass fraction of organic carbon present in the biochar (C_org_, Table 2); and *F*_*perm*_ indicates the fraction of that carbon remaining in the soil after a given period (in this study, a 100-year horizon was considered, with an average soil temperature of 25 °C). The value of *F*_*perm*_ is a function of the H/C_org_ ratio (Table 2) and was determined via Eq. (2), as proposed by Woolf et al.^32^.

4 $$\:{F}_{perm}=0.99-\:0.66\:\times\:\:\frac{H}{{C}_{org}}$$

## Results and discussion

The ultimate analysis revealed clear differences between the urban pruning waste and the derived biochars (Table 2). Urban pruning waste has a low C content (23.09%) and high H (3.29%), O (20.98%), and ash (51.60%) contents. It also presented elevated H/C (1.70) and O/C (0.68) ratios. After pyrolysis, all the biochars enriched with C and reduced the H and O contents (Table 2).

**Table 2:** Ultimate composition of the biochar samples and urban pruning waste.

Sample	Yield	Ash	C	H	*N*	O	H/C	O/C
			% ww^− 1^					
Urban pruning	–	51.60	23.09	3.29	0.94	20.98	1.70	0.68
BC1	28	17.18	71.63	2.06	0.82	13.60	0.34	0.14
BC2	44	29.03	56.01	2.50	1.19	18.06	0.53	0.24
BC3	32	38.61	47.38	2.59	1.52	19.69	0.65	0.31
BC4	31	15.40	70.80	2.40	0.02	11.39	0.40	0.12

Values are expressed as the means obtained from duplicates.

Among the samples, BC1 (C = 71.63%, H/C = 0.34, O/C = 0.14) and BC4 (C = 70.80%, H/C = 0.40, O/C = 0.12) showed the highest degrees of carbonization. Their low atomic ratios demonstrate a more advanced transformation of the organic matrix into condensed aromatic structures^33^. These characteristics are commonly linked with greater chemical stability and long-term persistence in soils^34^. On the other hand, BC3 presented the lowest C content (47.38%), together with the highest N content (1.52%) and ash content (38.61%). This composition suggests the presence of nitrogenous compounds in fine particles or the retention of volatiles, both of which are indicative of incomplete devolatilization. BC2 had intermediate values, but its high ash content (29.03%) points to mineral contamination^35,36^.

Importantly, the pyrolysis conditions (inlet temperature, average operating temperature, and residence time; Table 1) varied across the experiments. Consequently, the observed differences in biochar composition cannot be attributed to a single factor. However, under the evaluated conditions, the use of larger particles resulted in biochars with lower ash contents, indicating reduced mineral contamination of the feedstock. Among all the samples, BC4 had a high degree of carbonization, a low O/C ratio, and the lowest ash content. A reduced ash content improves the suitability of biochar for handling, transportation, and soil incorporation since it decreases the risk of windblown losses^37,38^. For these reasons, BC4 was selected as the most suitable biochar for subsequent investigations.

**Fig. 2:**
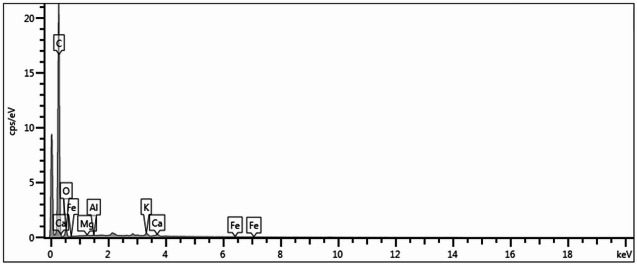
Energy-dispersive X-ray spectrum of biochar (BC4) from urban pruning waste.

**Fig. 3:**
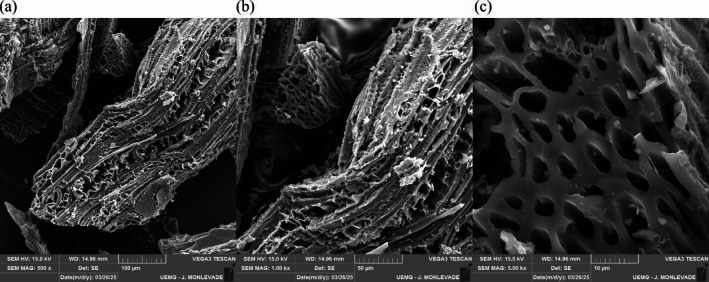
Micrographs of the biochar produced from urban pruning waste obtained in a continuous horizontal reactor at magnifications of (**a**) 500×, (**b**) 1000×, and (**c**) 5000×.

Given these considerations, the selected biochar (BC4) had an alkaline pH (8.77), a bulk density of 286.5 kg m^−3^, and moderate electrical conductivity (2.128 mS cm^−1^) (Table 3). These characteristics are consistent with the positive effects of biochar described in the literature, particularly its ability to neutralize soil acidity and improve the cation exchange capacity^39^. This is further supported by the levels of magnesium (Mg, 0.766 g kg^−1^), potassium (K, 4.802 g kg^−1^), and calcium (Ca, 9.942 g kg^−1^), whose presence contributes to the alkalinity and buffering capacity of biochar, which are desirable properties for soil acidity correction^40,41^. The presence of these elements was also confirmed by EDS, which primarily identified C, O, Mg, K, and Ca (Fig. 2). The porous structure of the biochar is also noteworthy, as evidenced by SEM micrographs showing a heterogeneous arrangement of elongated channels and interconnected cavities (Fig. 3a–c). This morphology favors water retention and highlights the structural complexity that enhances the potential of the material as a soil conditioner^42^.

**Table 3:** Characterization of the BC4 biochar sample produced from urban pruning waste via a continuous horizontal pyrolysis reactor.

Variable	Unit	Value	Limit values for class EBC-agro
Bulk density < 3 mm	kg m^-3^	286.5	–
pH in CaCl_2_	–	8.77	–
Electrical conductivity	mS cm^−1^	2.128	–
Salt content	mg KCl L^−1^	112.4	–
Organic carbon (C_org_)	%, w w^−1^	66.67	–
H/C_org_	–	0.45	< 0.7
O/C_org_	–	0.13	< 0.4
Total phosphorus (P)	g t^−1^	259.7	–
Iron (Fe)	g kg^−1^	3.803	
Potassium (K)	g kg^−1^	4.802	–
Magnesium (Mg)	g kg^−1^	0.766	–
Calcium (Ca)	g kg^−1^	9.942	–
Boron (B)	g t^−1^	22.8	–
Lead (Pb)	g t^−1^	< 1.0	120
Cadmium (Cd)	g t^−1^	< 1.0	1.5
Copper (Cu)	g t^−1^	8.7	100
Nickel (Ni)	g t^−1^	< 1.0	50
Mercury (Hg)	g t^−1^	< 10.0	1.0
Zinc (Zn)	g t^−1^	46.5	400
Chromium (Cr)	g t^−1^	2.3	90
Arsenic (Ar)	g t^−1^	< 10.0	13.0
Silver (Ag)	g t^−1^	< 1.0	–
Manganese (Mn)	g t^−1^	53.7	–
Acenaphthene	g t^−1^	< 0.02	–
Acenaphthylene	g t^−1^	< 0.02	–
Anthracene	g t^−1^	< 0.01	–
Benzo(a)anthracene	g t^−1^	< 0.02	–
Benzo(a)pyrene	g t^−1^	< 0.02	–
Benzo(b)fluoranthene	g t^−1^	< 0.02	–
Benzo(g, h,i)perylene	g t^−1^	< 0.02	–
Benzo(k)fluoranthene	g t^−1^	< 0.02	–
Chrysene	g t^−1^	< 0.05	–
Dibenzo(a, h)anthracene	g t^−1^	< 0.01	–
Phenanthrene	g t^−1^	< 0.02	–
Fluoranthene	g t^−1^	< 0.01	–
Fluorene	g t^−1^	< 0.03	–
Indeno(1,2,3-c, d)pyrene	g t^−1^	< 0.01	–
Naphthalene	g t^−1^	< 0.01	–
Pyrene	g t^−1^	< 0.03	–
Dioxins and furans	g t^−1^	< 0.01	–
Polychlorinated biphenyls (PCBs)	g t^−1^	< 0.001	0.02

The maximum limits established by the European Biochar Certificate (EBC) for polycyclic aromatic hydrocarbons (PAHs) in biocharare 6.0 ± 2.4 g t⁻¹ for the sum of the 16 PAHs defined by the United States Environmental Protection Agency (EPA) and 1.0 g t⁻¹ forthe sum of the 8 PAHs classified as carcinogenic by the European Food Safety Authority (EFSA).

**Fig. 4:**
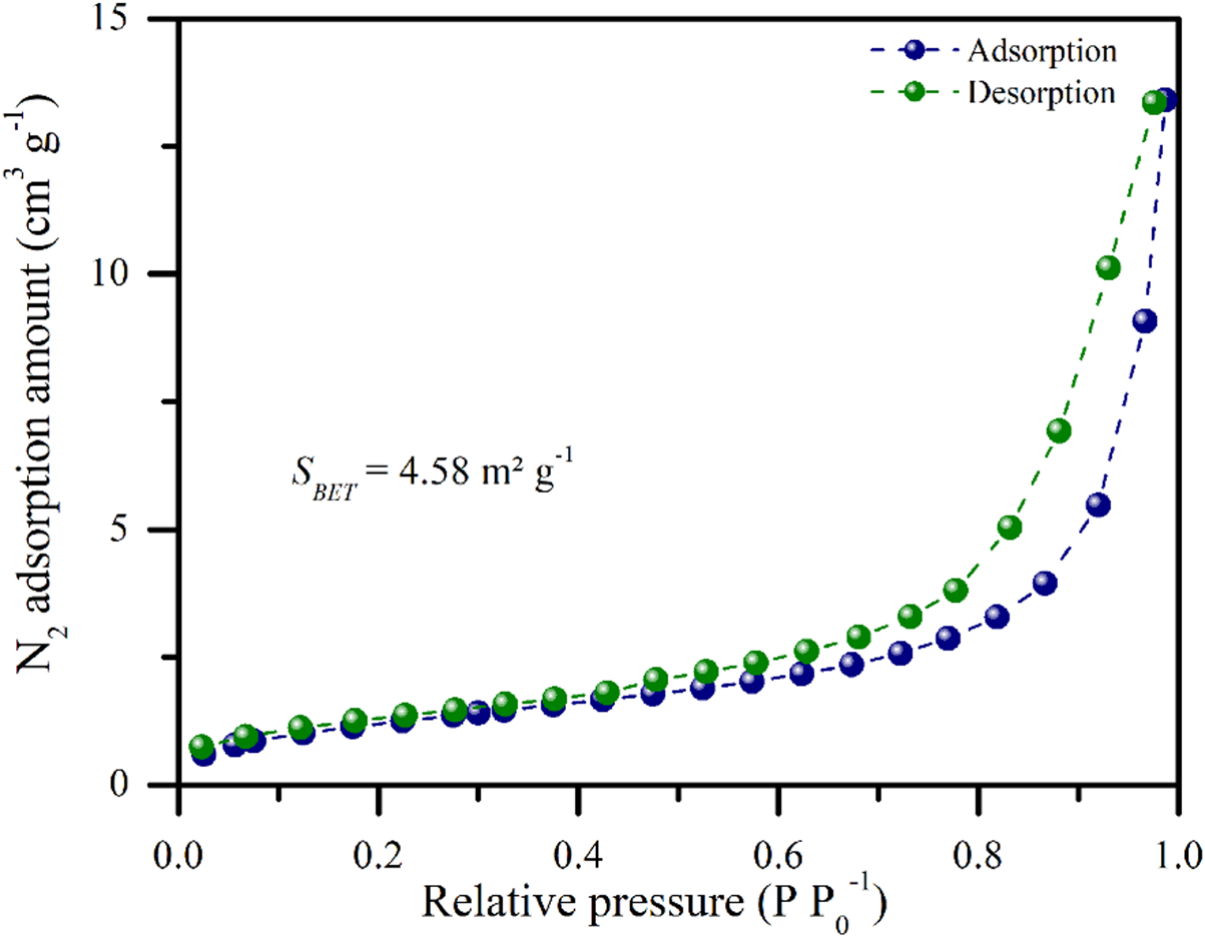
N_2_ adsorption‒desorption isotherms of biochar produced from urban pruning waste.

**Fig. 5:**
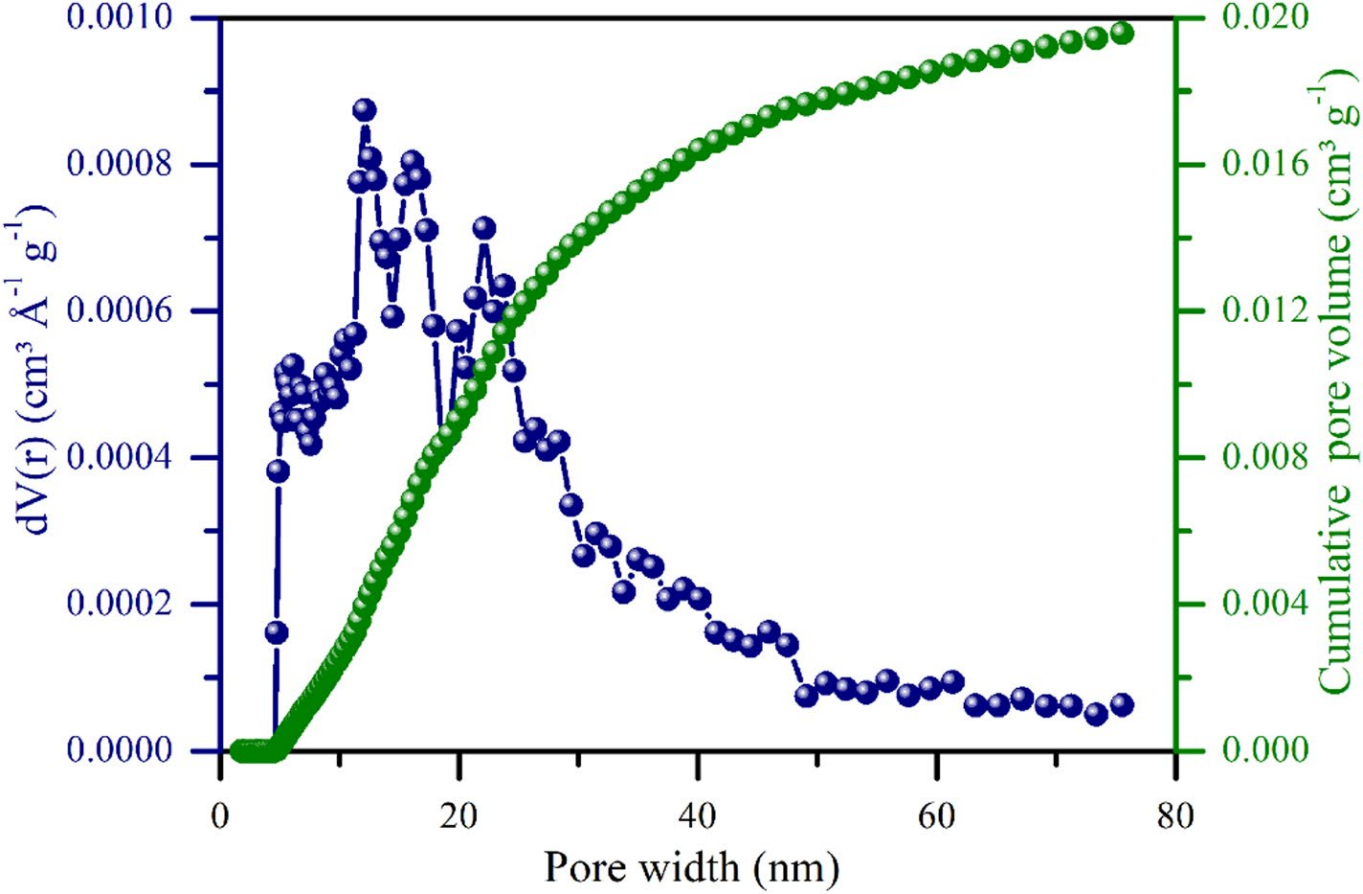
Pore size distribution and cumulative pore volume of biochar produced from urban pruning waste, which was calculated via the NLDFT model on the basis of N₂ adsorption data.

This structural characteristic was further supported by BET analysis, which revealed a specific surface area of 4.58 m² g^−1^ (Fig. 4), a total pore volume of 0.02 cm³ g⁻¹, and an average pore width of 12.12 nm (Fig. 5). According to the IUPAC classification, these values fall within the mesoporous range (2–50 nm)^31^, which is important for physicochemical interactions such as water retention and nutrient adsorption. On a different scale, SEM images (Fig. 3) also revealed larger pores in the micrometer range, which is consistent with those reported by Jaafar et al.^43^, who reported that pores of 20–100 μm act as preferential sites for fungal hyphal colonization. They also noted that while larger pores (~ 100 μm) facilitated hyphal attachment to pore walls, smaller pores (~ 20 μm) were often clogged by soil particles, restricting microbial access. Moreover, Hammer et al.^44^ demonstrated that arbuscular mycorrhizal fungi are able to explore even smaller pores (< 10 μm) within the biochar, which are inaccessible to most roots, thereby reinforcing the potential of this material to act as a reservoir of nutrients mobilized through fungal symbiosis.

In addition to its physical, chemical and morphological properties, the safety assessment of biochar is essential for its agricultural application. For this purpose, the contents of potentially toxic metals were determined (Table 3), and all analyzed elements presented concentrations below the limits established for the EBC-Feed and EBC-Agro classes^29^. Elements such as lead (< 1.0 g t^−1^), cadmium (< 0.1 g t^−1^), arsenic (< 10 g t^−1^), and mercury (< 0.1 g t^−1^) were below the limit of quantification, whereas copper (8.7 g t^−1^) and zinc (46.5 g t^−1^) remained below the maximum permissible values^29^. With respect to organic contaminants, all 16 polycyclic aromatic hydrocarbons (PAHs), as well as PCBs and dioxins, were also below the quantification limits (Table 3). These results demonstrate the compliance of the biochar sample with the parameters established by the EBC guidelines^29^, reinforcing its suitability for agricultural and environmental applications.

**Fig. 6:**
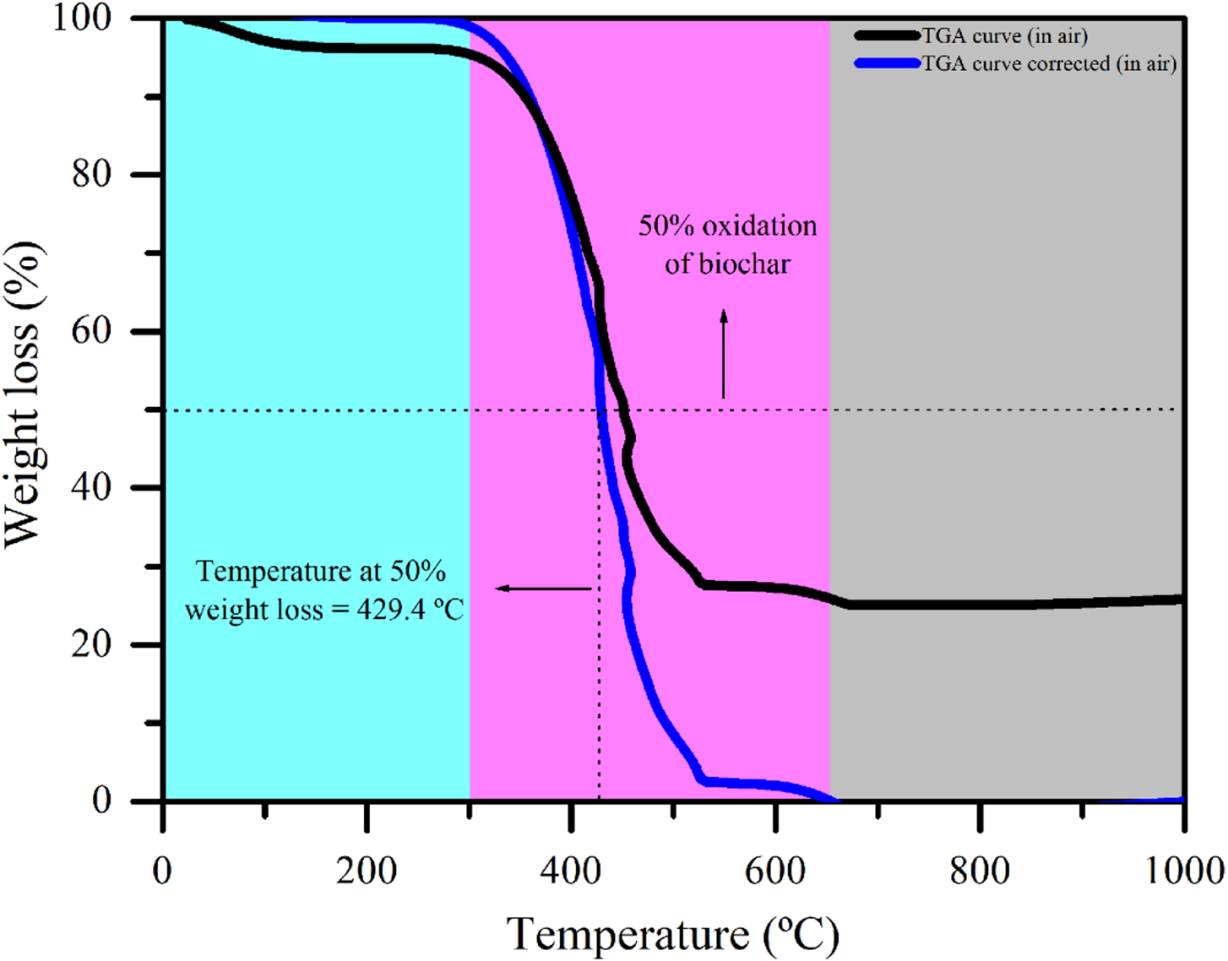
Thermogravimetric curves of biochar produced from urban pruning waste, obtained in a continuous horizontal reactor under a synthetic air atmosphere.

To complete the characterization of BC4, in the TGA (Fig. 6) under an oxidizing atmosphere (air), intense mass loss was observed from 400 °C, with 50% oxidation of the biochar occurring at 429.4 °C. This temperature was used for calculating the R_50_ index, which presented a value of 0.5, characterizing moderate stability according to the classification proposed by Li and Chen^44^. Higher R_50_ values indicate greater recalcitrance and, consequently, increased resistance to degradation. This index is classified into three categories: Class A (R_50_ ≥ 0.7), indicating low susceptibility to degradation; Class B (0.5 ≤ R_50_ < 0.7), denoting moderate stability; and Class C (R_50_ < 0.5), indicating high degradability. Therefore, the moderate stability observed in this study (R_50_ = 0.5) suggests that biochar may persist in soil for extended periods, contributing simultaneously to soil amendment and long-term climate change mitigation strategies.

The R_50_ index is a reliable proxy for predicting biochar persistence and carbon sequestration potential, as higher values are associated with increased resistance to chemical and biological oxidation and longer carbon residence times in soils^45^. Biochars with R_50_ values ≥ 0.7 are generally classified as highly stable, while values between 0.5 and 0.7, as observed for several biochars and composite materials, also indicate significant carbon sequestration potential^46,47^. Nonetheless, complementary indicators such as the H/C ratio may provide greater sensitivity to structural changes, since reductions in H/C values often reflect increased aromaticity and recalcitrance^48^. In this study, the atomic ratios of BC4 (H/C_org_ = 0.45; O/C_org_ = 0.13) fall below the recommended thresholds for agricultural application (H/C_org_ < 0.7; O/C_org_ < 0.4). The low O/C_org_ ratio also indicates a high stability, corresponding to an estimated half-life of 100–1000 years in soils^34^. Thus, the combined use of R_50_ and atomic ratios provides a robust framework for evaluating biochar stability and supports further discussion of its long-term role in carbon sequestration.

On the basis of the characterizations described above, BC4 has promising characteristics for agricultural and environmental applications, as its condensed aromatic structures contribute to its physicochemical stability. Nonetheless, agronomic performance still requires validation under field conditions, given the strong influence of soil type, crop species, application rates, and management practices on biochar efficacy. Another practical limitation concerns the adjustment of the particle size to meet agricultural input specifications, which was not addressed in this study. Even with these constraints, the findings provide relevant insights into how particle size can affect yield and composition while also confirming the strong carbon sequestration potential of BC4, thereby reinforcing its suitability for sustainable agricultural use and long-term climate change mitigation.

Among the most consistent benefits of biochar is its capacity for carbon sequestration. For BC4, the estimated potential was 1.62 t CO₂e t^−1^ of biochar (or 0.50 t CO₂e t^−1^ dry urban pruning), considering the fraction of stable carbon and a 100-year residence time at an average soil temperature of 25 °C. Although this approach follows widely adopted carbon accounting frameworks, uncertainties remain, as stability is affected by soil type, climate, erosion, mineralization, or even accidental combustion^49^. Life cycle assessments further indicate that the net greenhouse gas balance depends on the pyrolysis energy source, logistics, and application scale, with reported mitigation rates ranging between 0.4 and 1.2 t CO₂e t^−1^ dry feedstock^50^. In addition to carbon storage, co-benefits such as reductions in N_2_O and CH_4_ emissions^51^ may increase the overall mitigation efficiency of biochar compared with other strategies.

Overall, the biochar produced from urban pruning waste (BC4) had a stable composition, low ash content, and atomic ratios below the recommended limits for agricultural application, confirming its potential for persistence in soil. The estimated carbon sequestration value (1.62 t CO₂e t^− 1^ of biochar) falls within the range reported in previous studies, although methodological and scale differences account for some of the observed variation. For example, Yang et al.^52^ estimated up to 0.92 t CO₂e t^− 1^ dry feedstock, whereas Meng et al.^53^ demonstrated reductions of up to 1.47 t CO₂e t^− 1^ of crude steel when biochar was used to partially replace fossil fuels in the iron and steel industry. In agricultural scenarios, Lefebvre et al.^54^ projected mitigation rates between 3 and 5 t CO₂e ha^− 1^ over 20 years. These findings reinforce that although the magnitude of carbon sequestration varies depending on the application context and underlying assumptions, urban pruning waste biochar represents a robust climate change mitigation strategy that combines carbon stability with additional benefits to agricultural and environmental systems.

## Conclusion

Biochar produced from urban pruning waste in a continuous horizontal reactor demonstrated promising physicochemical characteristics, with a low ash content, atomic ratios within recommended thresholds, and compliance with EBC limits for potentially toxic metals and organic contaminants. The BC4 sample (PS < 90 mm) showed high recalcitrance according to the H/C_org_, O/C_org_, and R_50_ indices, indicating long-term persistence in soils. Furthermore, the estimated carbon sequestration potential (1.62 t CO₂e t^− 1^ of biochar) highlights the role of urban pruning waste biochar as a climate change mitigation strategy. However, the heterogeneity of the feedstock and the presence of soil and rock particles underscore the importance of prescreening and controlling particle size for obtaining high-quality biochar. In addition to these technical attributes, this study did not explore another key challenge associated with urban pruning, namely, the logistics of collection and transport to processing facilities. Therefore, we recommend that future studies combine agronomic field trials under different edaphoclimatic conditions with technoeconomic and logistical assessments to evaluate the scalability, environmental performance, and practical feasibility of incorporating urban pruning biochar into agricultural and environmental management strategies.

## Data Availability

The data presented in this study are available upon request from the corresponding authors.
